# Distinct Roles for COMPASS Core Subunits Set1, Trx, and Trr in the Epigenetic Regulation of *Drosophila* Heart Development

**DOI:** 10.3390/ijms242417314

**Published:** 2023-12-09

**Authors:** Jun-yi Zhu, Hangnoh Lee, Xiaohu Huang, Joyce van de Leemput, Zhe Han

**Affiliations:** 1 Center for Precision Disease Modeling, Department of Medicine, University of Maryland School of Medicine, Baltimore, MD 21201, USA; 2 Division of Endocrinology, Diabetes and Nutrition, Department of Medicine, University of Maryland School of Medicine, Baltimore, MD 21201, USA

**Keywords:** heart development, histone modification, *Drosophila*, *Set1*, *trx*, *trr*

## Abstract

Highly evolutionarily conserved multiprotein complexes termed Complex of Proteins Associated with Set1 (COMPASS) are required for histone 3 lysine 4 (H3K4) methylation. *Drosophila* Set1, Trx, and Trr form the core subunits of these complexes. We show that flies deficient in any of these three subunits demonstrated high lethality at eclosion (emergence of adult flies from their pupal cases) and significantly shortened lifespans for the adults that did emerge. Silencing *Set1*, *trx*, or *trr* in the heart led to a reduction in H3K4 monomethylation (H3K4me1) and dimethylation (H3K4me2), reflecting their distinct roles in H3K4 methylation. Furthermore, we studied the gene expression patterns regulated by Set1, Trx, and Trr. Each of the COMPASS core subunits controls the methylation of different sets of genes, with many metabolic pathways active early in development and throughout, while muscle and heart differentiation processes were methylated during later stages of development. Taken together, our findings demonstrate the roles of COMPASS series complex core subunits Set1, Trx, and Trr in regulating histone methylation during heart development and, given their implication in congenital heart diseases, inform research on heart disease.

## 1. Introduction

Histone modifications strongly influence gene expression levels in fetal and adult hearts [1,2,3,4]. In particular, the methylation status of histone H3 lysine 4 (H3K4) is strongly associated with gene expression activity through regulating transcription factor access to chromatin, transcriptional fidelity, and splicing outcomes [5]. These features are highly conserved in heart development from insects to mammals. In addition, mutations in histone-modifying genes are enriched, i.e., present more than expected by chance, among genes with de novo variants in patients with congenital heart disease [6].

A series of multiprotein complexes termed Complex of Proteins Associated with Set1 (COMPASS) are involved in H3K4 methylation [7]. Their components are greatly conserved from flies to mammals [7,8,9,10]. In mammals, the Lysine methyltransferase (KMT/Mixed Lineage Leukemia, MLL) family genes *KMT2A*, *KMT2B*, *KMT2C*, *KMT2D*, *SETD1A*, and *SETD1B* encode core subunits of COMPASS series complexes. All these core subunits share a Su(var)3–9, Enhancer-of-zeste, and Trithorax (SET) domain, which plays a fundamental role in the epigenetic regulation of gene expression [11,12]. Previous studies have implicated KMT family genes in many diverse cancer types [13,14]. More recently, de novo mutations in *KMT2A* in patients with congenital heart disease have been reported [15,16]. In addition, 18 mutations in *KMT2C* have been identified in patients with congenital heart disease [17]. In line with the patient data, a mouse model in which the KMT2C SET domain was deleted, showed abnormal heart development and ventricular septal defects [17]. Finally, KMT2D has been shown to regulate cardiac gene expression during heart development primarily via H3K4 dimethylation [18]. Indeed, loss of *KMT2D* led to significant heart defects in both mice and zebrafish [18,19,20,21]. In addition, mutations in *KMT2D* have been associated with Kabuki syndrome, a congenital disorder marked by multiple malformations, the most prominent of which are distinctive craniofacial features [22]. Of the patients with mutations in *KMT2D*, 70% presented with congenital heart disease [23]. These studies demonstrate the importance of KMT family members in heart development and their association with congenital heart disease. However, the exact roles of these COMPASS core subunits in heart development remain unclear, as does whether the other KMT family members (KMT2B, SETD1A, and SETD1B) play equally important roles during heart development.

To gain further insight into the role of KMT family members in cardiac development, we used *Drosophila*, an established model system to study heart development and disease [24]. In fact, we previously used a *Drosophila* high-throughput in vivo functional screen to validate candidate genes identified from congenital heart disease patient genomic sequencing [25]. We found that heart-specific silencing of histone-modifying genes, including H3K4 methylases, led to heart morphological and functional defects [25]. Three COMPASS series complexes are known for *Drosophila*: Set1/COMPASS, Trithorax COMPASS-like, and Trithorax-related COMPASS-like. Each of their core subunits—Set1, Trx, and Trr—conveys H3K4 methyltransferase activity [7,8]. Recently, we showed that fly Lpt and Trr function together, equivalent to mammalian KMT2C and KMT2D, in H3K4 methylation that regulates transcription during heart development [10]. Here, we looked even earlier in development and show that cardiac-specific silencing of the core subunits of the *Drosophila* COMPASS series complex disrupted methylation patterns in cardiomyocytes and led to structural and functional cardiac defects. We studied fly *SET domain containing 1* (*Set1*), the homolog of human *SET1DA* and *SETD1B*; fly *trithorax* (*trx*), the homolog of human *KMT2A* and *KMT2B*; and fly *trithorax-related* (*trr*), the homolog of human *KMT2C* and *KMT2D*. We combined the structural and functional heart outcomes with transcriptomics following *Set1*, *trx*, or *trr* silencing in the fly heart. The data indicate distinct roles for Set1, Trx, and Trr in the regulation of histone methylation at specific times (early vs. late) during fly heart development.

## 2. Results

### 2.1. Temporal Expression Patterns of Set1, Trx, and Trr across Embryonic Heart Developmental Stages in Flies

**Figure 1:**
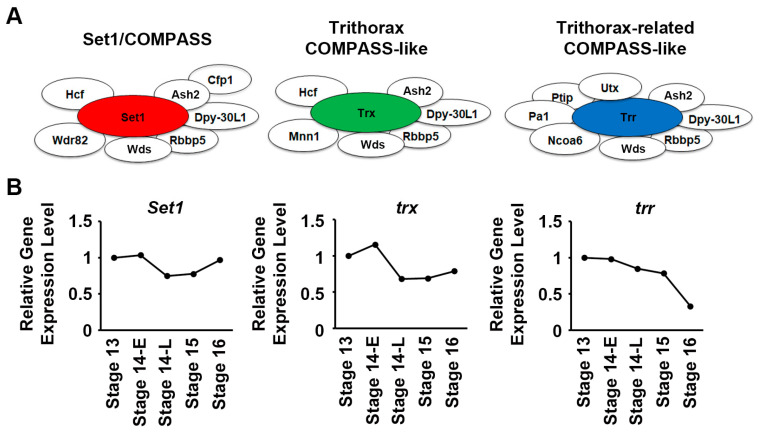
*Drosophila* COMPASS core *Set1*, *trx*, and *trr* expression at different embryonic heart developmental stages. (**A**) Schematic representation of three different *Drosophila* COMPASS complexes: Set1/COMPASS, Trithorax (Trx) COMPASS-like, and Trithorax-related (Trr) COMPASS-like complex. Ash2, Absent, small, or homeotic discs 2; Cfp1, CXXC finger protein 1; Dpy-30L1, Dpy-30-like 1; Hcf, Host cell factor; Mnn1, Menin 1; Ncoa6, Nuclear receptor coactivator 6; Pa1, PTIP associated 1; Ptip, PAX transcription activation domain interacting protein; Rbbp5, Retinoblastoma binding protein 5; Set1, SET domain containing 1; Utx, Utx histone demethylase; Wdr82, WD repeat domain 82; Wds, Will die slowly. (**B**) Relative gene expression levels of *Set1*, *trx*, and *trr* in *Drosophila* heart (cardiogenic progenitors) at embryonic stages 13, 14-E (early), 14-L (late), 15, and 16. None of the changes reached statistical significance.

The COMPASS series complexes share many components, and at their core, each has a histone H3K4 methyltransferase subunit that marks the complex. In *Drosophila,* these are Set1/COMPASS, Trithorax COMPASS-like, and Trithorax-related COMPASS-like (Figure 1A). To gain knowledge about the role of these COMPASS complexes, we first looked at the expression levels of the genes encoding these core H3K4 methyltransferases in cardiac progenitor cells from our single-cell RNAseq data [26]. Cells were collected during critical developmental stages of *Drosophila* heart development; from the migration of bilateral rows of cardiac progenitors (stage 13 onward) to the formation of a closed heart tube (stage 16). *Set1* and *trx* showed steady expression levels throughout development; however, while not quite reaching significance, *trr* showed a 30–40% drop in expression by stage 16 (Figure 1B). This raises the question of whether this minor shift in *trr* expression signifies any functional changes at the cellular level.

### 2.2. Set1, Trx, and Trr Silencing Impacted Larval Heart Development at Different Larval Stages

**Figure 2:**
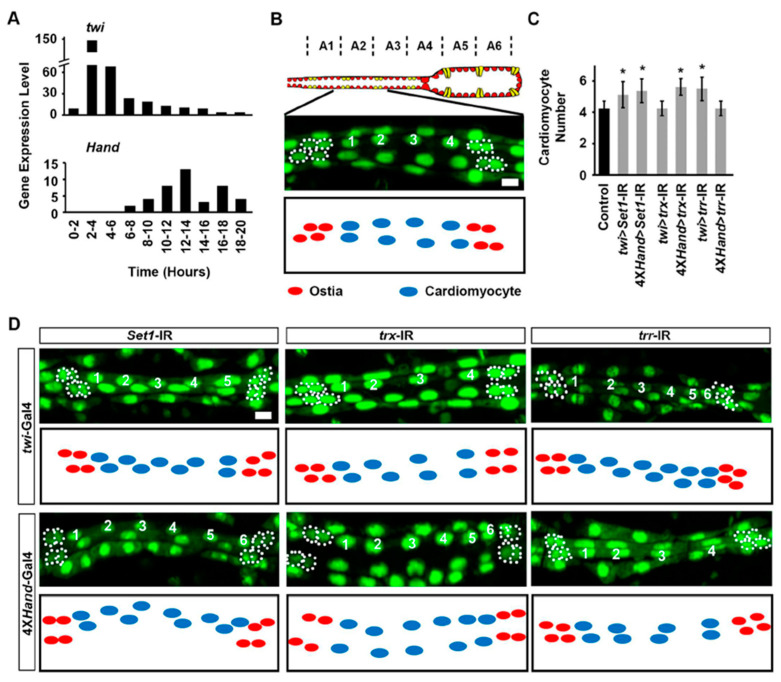
Heart development at early and late fly larval developmental stages following heart-specific silencing of *Set1*, *trx*, or *trr*. (**A**) Gene expression levels for *twist* (*twi*) and *Hand* (*Hand*) at different times (hours) after egg laying, in whole larva. Data from the modENCODE_mRNA-Seq_development project. (**B**) Representative live image of 1st instar *Drosophila* larva heart (*Hand*-GFP;*twi*-Gal4^+/−^). *Hand*-GFP expression (green; nuclear) labels the cardiomyocytes (numbered 1–4), ostia (outlined by dashed line), and pericardial cells. Typically, each hemisegment contains four cardiomyocytes and two ostia. Graphic depiction below. Scale bar = 20 µm. (**C**) Quantitation of larval heart cardiomyocyte number in one hemisegment (see representative images in (**D**)). Control, *Hand*-GFP;*twi*-Gal4^+/−^; all RNAi (-IR) lines also expressed *Hand*-GFP. *n* = 6 larvae per genotype. Values are presented as mean along with the standard deviation (s.d). Statistical significance (*) was defined as *p* < 0.05 using Kruskal–Wallis H-test followed by Dunn’s test. (**D**) Live images of heart for larvae expressing *twi*-Gal4 and 4X*Hand*-Gal4 of UAS-RNAi transgenes targeting *Set1*, *trx* or *trr*, *Set1*-IR, *trx*-IR or *trr*-IR (IR, Interference of RNA). Ostia are outlined by dashed lines and cardiomyocytes are indicated by a number. All RNAi (-IR) lines also expressed *Hand*-GFP, which labels heart cell nuclei (green). Scale bar = 20 µm.

**Figure 3:**
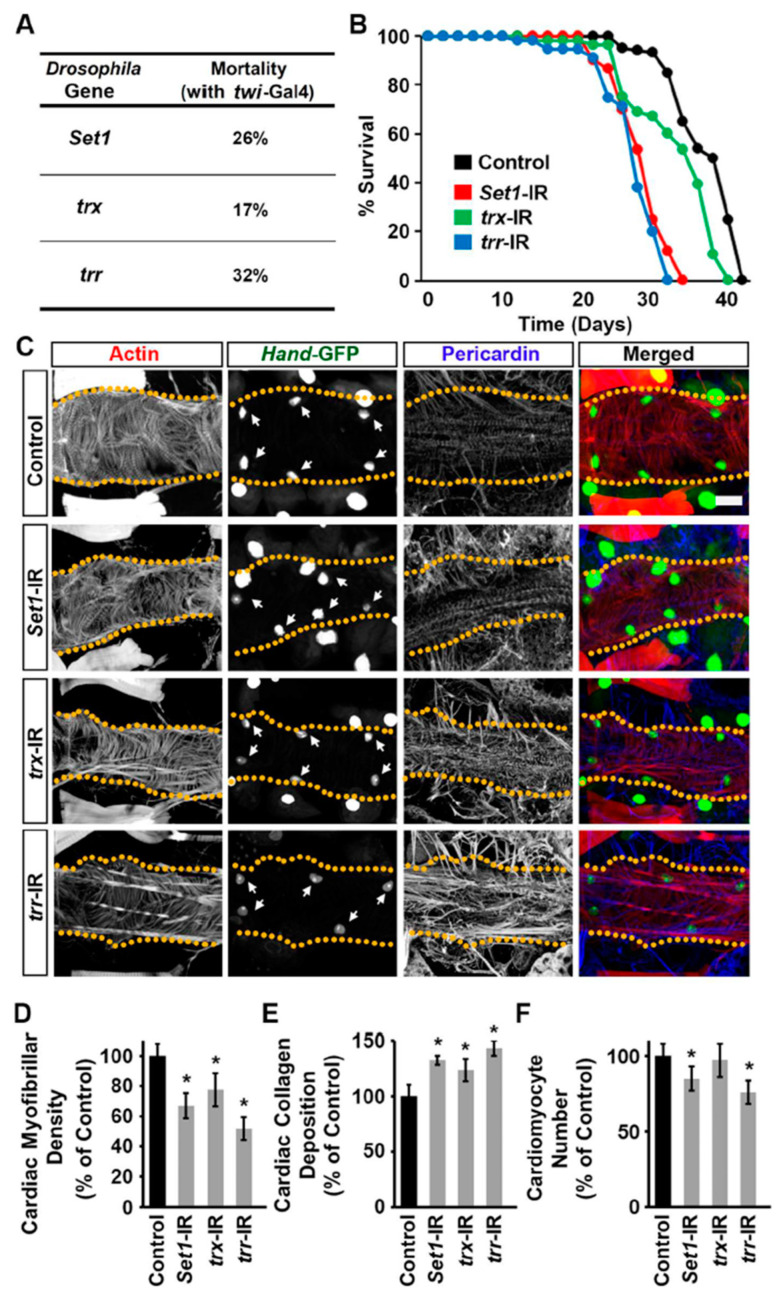
Heart structure and pericardin deposition in flies following heart-specific silencing of *Set1*, *trx*, or *trr*. (**A**) Eclosion lethality induced by *twi*-Gal4-driven expression of UAS-RNAi transgenes targeting *Set1*, *trx*, or *trr* starting from the early larval fly developmental stage. By crossing with a CyO (curly wing) balancer, emerging adults with curly wings (CyO) have no transgene expression, whereas those with straight wings express *twi*-Gal4 > RNAi. The mortality rate is calculated as [((curly—straight)/curly) × 100]. All lines also expressed *Hand*-Gal4. *n* = 400+ flies (female and male) per genotype. (**B**) Survival curves for adult flies expressing *Set1*, *trx*, or *trr* RNAi (-IR) transgenes (*twi*-Gal4) in heart cells; and *Hand*-GFP. Control, *Hand*-GFP;*twi*-Gal4^+/−^. *n* = 100 male flies (20 flies/vial) per genotype. (**C**) Adult (5-day-old females) heart phenotype induced by expression of UAS-RNAi transgenes targeting *Set1*, *trx*, or *trr* starting from the early larval developmental stage (*twi*-Gal4). Cardiac actin myofibers were visualized by phalloidin staining (red). *Hand*-GFP expression (green; nuclear) labels cardiomyocytes (heart cells). Pericardin was detected by immunofluorescence (blue). Dotted lines delineate the outline of the heart tube. Arrows point to heart cardiomyocytes. Control, *Hand*-GFP;*twi*-Gal4^+/−^. Scale bar = 40 µm. (**D**) Quantitation of adult heart cardiac myofibrillar density relative to control (see (**C**)). *n* = 6 flies (5-day-old females) per genotype. (**E**) Quantitation of adult heart cardiac collagen (Pericardin) deposition relative to control (see (**C**)). *n* = 6 flies (5-day-old females) per genotype. (**F**) Quantitation of adult heart cardiomyocyte numbers relative to control (see **C**). *n* = 6 flies (5-day-old females) per genotype. Values are presented as mean along with the standard deviation (s.d). Statistical significance (*) was defined as *p* < 0.05 using Kruskal–Wallis H-test followed by Dunn’s test.

**Figure 4:**
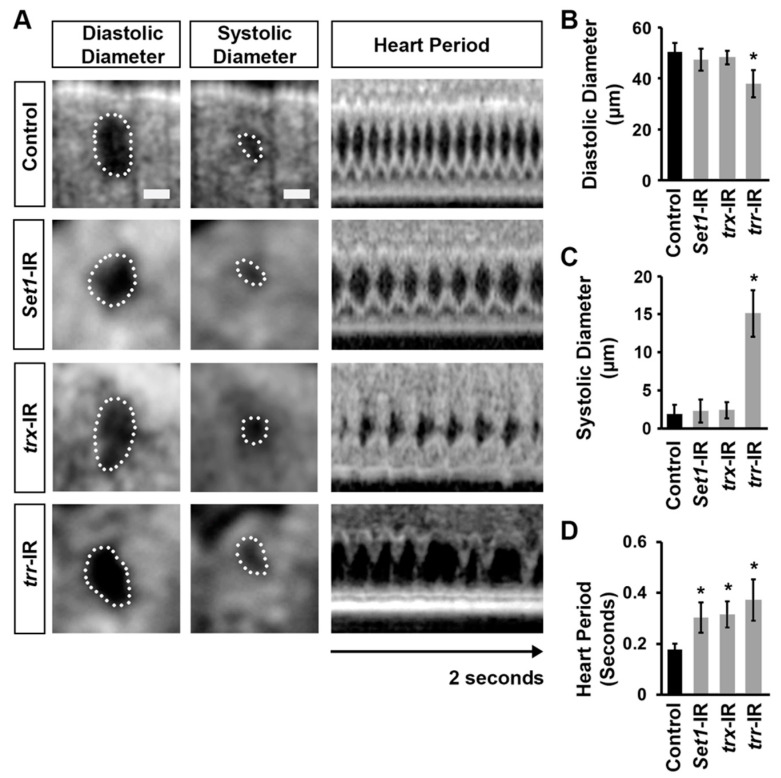
Cardiac function in flies following heart-specific silencing of *Set1*, *trx*, or *trr*. (**A**) Images from *Drosophila* (4-day-old females) heartbeat videos obtained by optical coherence tomography (OCT). Representative images show changes in heart function induced by expression of UAS-RNAi transgenes targeting *Set1*, *trx*, or *trr* starting from the early larval developmental stage (*twi*-Gal4). Control, *Hand*-GFP;*twi*-Gal4^+/−^; all RNAi (-IR) lines also expressed *Hand*-GFP. Dotted line outlines the circumference of the heart tube. Scare bar = 20 µm. (**B**) Quantitation of adult heart diastolic diameter. *n* = 10 flies (4-day-old females) per genotype (see (**A**)). (**C**) Quantitation of adult heart systolic diameter. *n* = 10 flies (4-day-old females) per genotype (see (**A**)). (**D**) Quantitation of heart period. *n* = 10 flies (4-day-old females) per genotype (see (**A**)). Values are presented as mean along with the standard deviation (s.d). Statistical significance (*) was defined as *p* < 0.05 using Kruskal–Wallis H-test followed by Dunn’s test.

**Figure 5:**
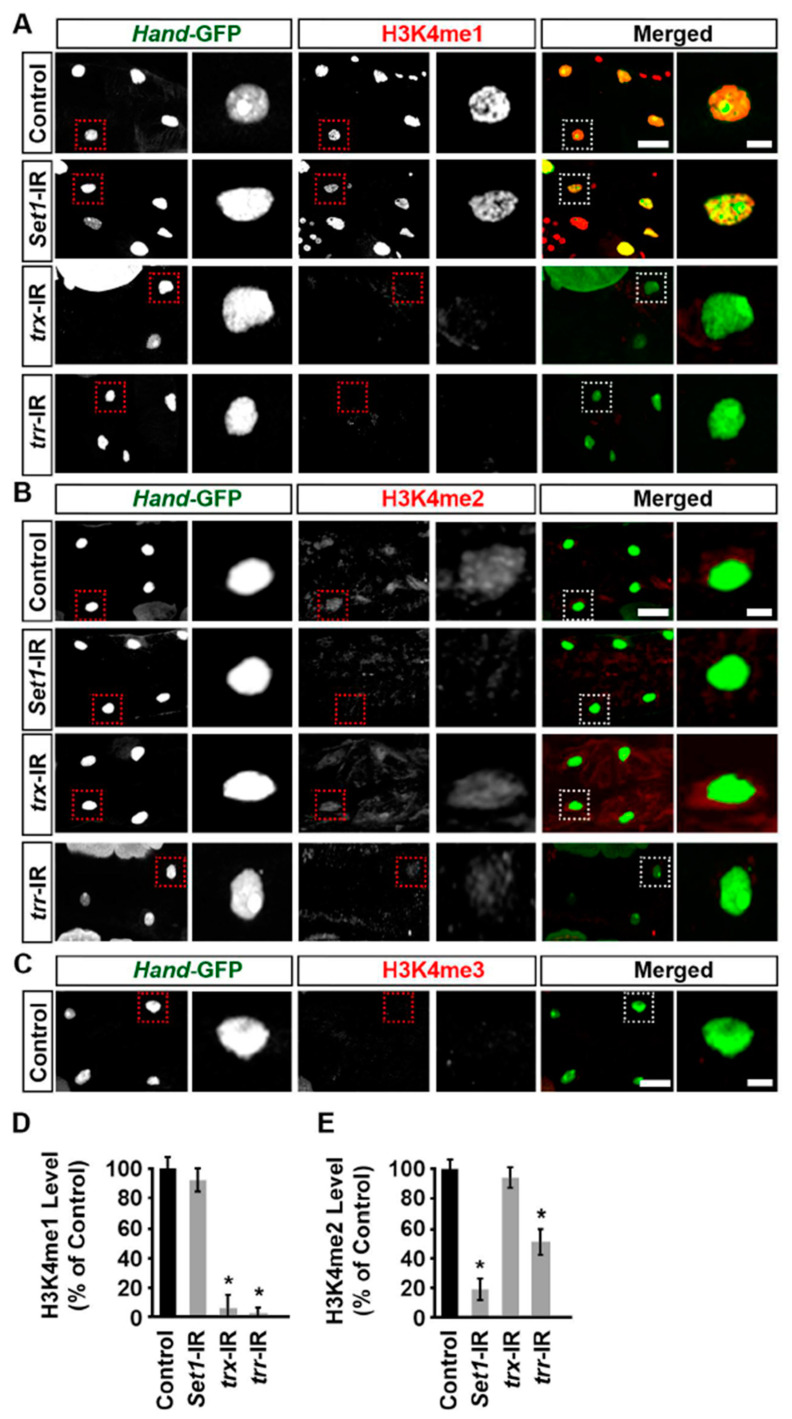
H3K4 methylation levels in cardiomyocytes of heart-specific *Set1*-, *trx*-, or *trr*-RNAi flies. (**A**–**C**) Representative images for adult heart H3K4 methylation: (**A**) monomethylation (H3K4me1); (**B**) dimethylation (H3K4me2); and (**C**) trimethylation (H3K4me3). Flies (5-day-old females) expressed UAS-RNAi transgenes targeting *Set1*, *trx*, or *trr* driven by (*twi*-Gal4), starting from the early larval fly developmental stage. Control, *Hand*-GFP;*twi*-Gal4^+/−^; all RNAi (-IR) lines also expressed *Hand*-GFP. The dotted box indicates the magnified area to show the cardiomyocyte nucleus. Scale bar = 20 μm. Scale bar (magnification) = 5 μm. (**D**) Quantitation of adult heart cardiac H3K4me1 level relative to the level in control flies (*Hand*-GFP;*twi*-Gal4^+/−^; 5-day-old females). *n* = 24 cardiomyocytes (six flies, four cardiomyocytes each) per genotype (see (**A**–**C**)). (**E**) Quantitation of adult heart cardiac H3K4me2 level relative to the level in control flies (*Hand*-GFP;*twi*-Gal4^+/−^; 5-day-old females). *n* = 24 cardiomyocytes (six flies, four cardiomyocytes each) per genotype (see (**A**–**C**)). Values are presented as mean along with the standard deviation (s.d). Statistical significance (*) was defined as *p* < 0.05 using Kruskal–Wallis H-test followed by Dunn’s test.

**Figure 6:**
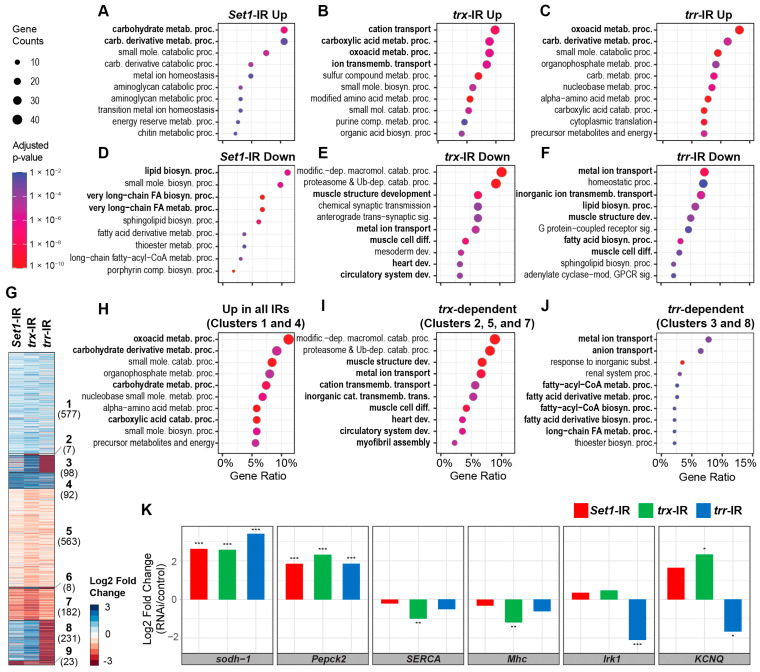
Differential cardiac transcriptomic responses in heart-specific *Set1*-, *trx*-, or *trr*-RNAi flies. Flies (one-week-old females): *Set1*-IR (*Hand*-GFP;*twi*-Gal4 > *Set1*-RNAi), *trr*-IR (*Hand*-GFP;*twi*-Gal4 > *trr*-RNAi), *trx*-IR (*Hand*-GFP;*twi*-Gal4 > *trx*-RNAi). Each sample contained RNA extracted from 50 dissected fly hearts. (**A**–**F**) Enriched functional terms from Gene Ontology (GO) analysis for the significantly upregulated (**A**–**C**) or downregulated (**D**–**F**) genes for flies with *Set1* (**A**,**D**), *trx* (**B**,**E**), or *trr* (**C**,**F**) silenced in the heart. (**G**) A heatmap (log2 scale) summarizes the differential expression analyses between *Set1-*, *trx-*, and *trr*-silenced hearts, compared to control (*Hand*-GFP;*twi*-Gal4^+/−^). Gene clusters were obtained by *k*-means clustering; cluster name in bold text, number of genes in cluster in brackets. Genes that did not show significant differential expression (adjusted *p*-value < 0.05) from any of the comparisons or that were not expressed in any of the samples (Transcripts per million < 1) were omitted. (**H**–**J**) Enriched GO functional terms for genes grouped by similar differential expression patterns across *Set1*-IR, *trx*-IR, and *trr*-IR fly hearts. The indicated clusters correspond to the heatmap (see **G**). (**H**) Enriched GO terms for gene clusters that represent negative regulation by *Set1*, *trx*, and *trr*, i.e., upregulated expression in all three IR lines. (**I**) Clusters that are more dependent on *trx*, i.e., more downregulated in *trx*-IR than in *trr*-IR fly hearts. (**J**) Clusters with *trr*-specific regulation, i.e., that show downregulation in *trr*-IR but upregulation in *trx*-IR. (**K**) Graphs plot differentially expressed genes from the enriched GO terms in H-J: (**H**) *sodh-1* and *Pepck2*, metabolic genes; (**I**) *SERCA*, Ca^2+^ physiology, and *Mhc*, muscle-related genes; and (**J**) *Irk1* and *KCNQ*, ion transport genes. Statistical significance was defined as adjusted *p*-value < 0.05 using the Wald test and corrected for multiple testing using the Benjamini–Hochberg method; (*) signifies adjusted *p* < 0.05, (**) signifies adjusted *p* < 0.005, and (***) signifies adjusted *p* < 0.001.

To test whether there are functional or preferential changes in *Set1*, *trx*, and *trr* during fly heart development, we employed the *Drosophila* UAS-Gal4 system combined with RNAi knockdown. We combined either *twist* (*twi*)-Gal4 or 4X*Hand*-Gal4 (four tandem repeats of *Hand* [25]) with UAS-*Set1*-RNAi, UAS-*trx*-RNAi, or UAS-*trr*-RNAi. Data from the modENCODE_mRNA-Seq_development project available through FlyBase (accessions: NCBI BioProject, PRJNA75285; NCBI Sequence Read Archive, SRP001065; modENCODE_574) [27,28] showed that *twi* is expressed in the heart during early larval stages, 1st instar, whereas *Hand* is expressed in the heart during late larval stage; heart-specific (Figure 2A). Thus, this creates fly progenies with targeted RNAi-based silencing of *Set1*, *trx*, or *trr*, driven by Gal4 at different heart developmental stages. Two independent RNAi lines were tested for each gene and showed similar results; therefore, representative data for one line have been displayed in the figures (in Figure 2, Figure 3, Figure 4, Figure 5 and Figure 6, data have been shown for *Set1*-IR ID 40931, *trx*-IR ID 33703, and *trr*-IR ID 36916).

We observed that silencing *Set1* and *trr*, but not *trx*, early (*twi*-Gal4) led to increased cardiomyocyte numbers compared to the control; while silencing them late (4X*Hand*-Gal4) resulted in increased cardiomyocytes in *Set1* and *trx*, but not *trr*, compared to control in 4-day-old adult flies (Figure 2B–D). These results suggest that *Set1* is required to maintain cardiomyocyte numbers both early and later during larval development, whereas *trx* (late) and *trr* (early) are necessary during specific larval developmental stages only.

### 2.3. Set1, Trx, and Trr Silencing Impacted Survival and Cardiac Defects in Adult Drosophila

Previously, we had shown that silencing *trx* with 4X*Hand*-Gal4 results in the absence of a heart tube and leads to early lethality with no adult flies surviving beyond 25 days [25]. Therefore, we used *twi*-Gal4 to silence the COMPASS histone H3K4 methyltransferases to study their effect on the adult fly heart. When silencing *Set1*, *trx*, or *trr* driven by *twi*-Gal4, we observed high mortality at eclosion (i.e., emergence of adult flies from their pupal case): 26%, 17%, and 32%, respectively (Figure 3A). Adult flies that did emerge from these RNAi lines showed markedly shortened lifespans compared to control flies (*twi*-Gal4^+/−^), less so for *trx*-RNAi (Figure 3B).

To look at the effect on the adult fly heart structure, the hearts were stained with phalloidin to visualize the cardiac actin filaments. *Set1*, *trx*, or *trr* gene silencing (*twi*-Gal4 driver) in the heart was associated with overall disorganization of cardiac actin filaments and reduced cardiac muscle fiber density (Figure 3C,D). We also observed increased deposition of Pericardin, a type IV collagen that plays a critical role in maintaining *Drosophila* cardiac tissue integrity (Figure 3C,E). The overabundance of Pericardin indicates a pathophysiological condition of fibrosis and can be used as an indicator of cardiac injury [29,30,31,32]. Cardiomyocytes were marked by the expression of green fluorescent protein (GFP) driven by a *Hand* gene regulatory element (Figure 3C). Silencing *Set1* or *trr* led to significantly reduced cardiomyocyte numbers in the adult flies that survived (Figure 3C,F). Notably, at the larval stage, cardiomyocyte levels following *Set1* or *trr* silencing were increased (Figure 2C). We hypothesize that early-stage cardiomyocytes are dysfunctional and have died by the adult stage, resulting in reduced heart cell numbers (Figure 3F). Additional studies across the early developmental stages could confirm this. Together, the findings show that all three COMPASS core subunits are needed for a normal heart structure.

### 2.4. Set1, Trx, and Trr Silencing Induced Cardiac Dysfunction

To assess cardiac functional defects induced by silencing *Set1*, *trx*, or *trr* (*twi*-Gal4), we applied optical coherence tomography (OCT). The orthogonal view of the heart provides accurate and real-time measurements of the heart tube diameter and heart period (Figure 4A). Silencing of *trr*, but not *Set1* or *trx*, was associated with reduced diastolic and increased systolic diameters (Figure 4B,C). Compared to control flies (*twi*-Gal4^+/−^), the heart period in *Set1*, *trx*, and *trr* gene-silenced flies was significantly increased (Figure 4D). Thus, all three COMPASS core subunits are required for normal heart function.

### 2.5. Set1, Trx, and Trr Regulated H3K4 Mono- and Dimethylation Levels in the Drosophila Heart

Set1, Trx, and Trr have been shown to be essential in regulating gene expression through H3K4 methylation at enhancers and promoters [8]. To test whether they are similarly important for H3K4 methylation in the *Drosophila* heart, we examined H3K4 methylation status in the adult fly heart using immunofluorescence. In typical control flies, H3K4me1 and H3K4me2 were found in the nucleus of cardiomyocytes (Figure 5A,B), but we were unable to detect H3K4me3 (Figure 5C). We found that silencing *Set1* led to a significant reduction in H3K4me2 but not H3K4me1 levels in *Drosophila* cardiomyocyte nuclei (Figure 5A,B,D,E). On the other hand, silencing *trx* led to a significant reduction in H3K4me1 but not H3K4me2 levels, whereas silencing *trr* led to significant reductions in both the H3K4me1 and H3K4me2 levels in *Drosophila* cardiomyocyte nuclei (Figure 5A,B,D,E). These findings indicate that *Set1*, *trx*, and *trr* differentially regulate H3K4 methylation, which is crucial for *Drosophila* heart development.

### 2.6. Silencing Set1, Trx, or Trr Led to Differential Transcriptomic Responses in the Drosophila Heart

Next, we investigated the transcriptional control exerted by Set1, Trx, and Trr. RNAi-mediated silencing of each of the three COMPASS core subunits resulted in both shared and different transcriptomic responses in the adult *Drosophila* hearts (Figure 6A–F). For example, silencing *Set1* resulted in the upregulation of genes that are involved in the carbohydrate metabolic process (GO:0005975; adjusted *p*-value = 2.7 × 10^−7^. Fisher’s exact test, corrected with the Benjamini-Hochberg method) (Figure 6A) and the downregulation of lipid biosynthetic process (GO:0008610)-related genes (adjusted *p*-value = 7.16 × 10^−7^) (Figure 6D). Silencing either *trx* or *trr* led to increased expression of oxoacid metabolic process genes (GO:0043436, adjusted *p* values 7.49 × 10^−7^ and, 1.46 × 10^−12^, respectively) (Figure 6B,C). Interestingly, we observed that ion transport-related genes (GO:0034220 and GO:0030001) were significantly enriched among the upregulated genes in the *trx*-silenced hearts (adjusted *p*-value = 1.34 × 10^−6^) (Figure 6B), as well as among the downregulated genes in the *trr* silenced hearts (adjusted *p*-value = 9.8 × 10^−9^) (Figure 6F). Silencing either *trx* or *trr* led to the downregulation of muscle development-related genes (GO:0061061; adjusted *p* values = 3.94 × 10^−8^, and 0.00016, respectively) (Figure 6E,F).

To gain further insight into the regulation by each COMPASS core subunit, we performed *k*-means clustering analysis on the differential expression profiles (Figure 6G). The data revealed diverse transcriptional programs, some of which were controlled by all three KMTs, *Set1*, *trx*, and *trr* (Figure 6H). The genes that demonstrated increased expression following knockdown of either COMPASS KMT included metabolic genes that are required for carboxylic acid (GO:0019752; adj. *p* = 3.04 × 10^−16^) or oxoacid metabolism (GO:0043436; adj. *p* = 1.54 × 10^−17^) pathways (Figure 6H). These GO terms include the fly *sorbitol dehydrogenase 1* (*sodh-1*) and *Phosphoenolpyruvate carboxykinase* (*Pepck2*) genes (Figure 6K).

Given the differences in temporal expression for *trx* and *trr* during heart development in the fly (Figure 1 and Figure 2), we tested if this corresponded to differential regulation of gene expression. While Trx and Trr shared many common target genes, we also found genes that showed a greater reduction in expression in *trx*-silenced fly hearts (i.e., *trx*-dependent activation; Figure 6I). These genes were enriched for functional categories of muscle structure development (GO:0061061; adj. *p* = 1.88 × 10^−11^), metal ion transport (GO:0030001; adj. *p* = 3.52 × 10^−9^), and cation transmembrane transport (GO:0098655; adj. *p* = 1.41 × 10^−5^). Genes in these categories included *SERCA* for Ca^2+^ physiology and *Mhc* for muscle structure GO terms (Figure 6K).

Other gene clusters showed significantly decreased expression in *trr*-IR fly hearts but increased expression in *trx*-IR fly hearts (Figure 6J). This suggests that these genes are targets, direct or indirect, of *trr*-mediated transactivation at the later stages of heart development (i.e., *trr*-dependent). These clusters included genes required for metal and/or anion transports (GO:0030001 and GO:0006820, adjusted *p*-value = 0.0038 for both), which are essential for muscle maturation. They included the *Drosophila* ortholog of the human potassium channel gene *KCNJ2* (*Irk1*; Figure 6K)—the *KCNJ2* mutation has been associated with long QT syndrome in human hearts [33]—as well as a voltage-gated channel gene (*KCNQ*; Figure 6K) that has been known to function in cardiac muscle contraction [34]. In contrast, we found only seven genes with *trx*-specific upregulation (i.e., decreased expression only in *trx*-IR fly hearts; Figure 6G, Cluster 2). While these genes did not show any enriched functional terms in our GO analysis, we did find that six out of the seven genes are known targets of the *Drosophila p53* transcription factor (adjusted *p*-value = 8.13 × 10^−9^) [35].

These data suggest that each of the COMPASS core subunits controls histone methylation of distinct sets of genes, with many metabolic pathways active early in development and throughout, while cardiac development and Ca^2+^ physiology processes are active during later stages of development.

## 3. Discussion

### 3.1. Set1, Trx, and Trr Distinctly Contribute to Methylation during Drosophila Heart Development

*Drosophila* Set1 is homologous to the mammalian SETD1A and SETD1B COMPASS core subunits, which display mono-, di-, and trimethyltransferase (H3K4me1, -me2, and -me3) activity [36,37,38]. However, their activity in the heart and during its development remains unknown. Here, we demonstrate that in the developing fly heart, Set1 displays H3K4me2 activity to regulate gene expression patterns that promote healthy heart development.

Fly Trx is homologous to mammalian KMT2A and KMT2B. Mutations in *KMT2A* cause Wiedemann–Steiner syndrome, a rare congenital disease with multiple anomalies [39,40]. Around 35% of patients present with cardiac abnormalities, mostly structural [41]. We had previously shown that *trx*-deficiency in the fly heart (4X*Hand* > *trx*-RNAi) resulted in the absence of a heart structure [25]. Here, using a different driver, we demonstrate that Trx induces H3K4me1 activity to regulate gene expression in the developing fly heart.

We recently showed that Lpt and Trr work together to conduct the function of mammalian KMT2C and KMT2D in regulating methylation and transcription during heart development [10]. Whereas for the previous study, we had used 4X*hand*-Gal4 to drive heart-specific silencing of *Lpt* and *trr*, here we used *twi*-Gal4, which is expressed earlier during development, to silence the genes encoding the COMPASS core methyltransferases. With either driver, silencing *trr* in the developing heart led to the same structural and functional cardiac phenotypes. In addition, in patients and a mouse model, increased KMT2C mRNA and protein levels were linked to dilated cardiomyopathy [42]. Moreover, H3K4me2, but not -me3, levels were increased in heart tissue from patients with dilated cardiomyopathy [42]. The study did not test H3K4me1 levels, even though KMT2C H3K4me1 and -me2 were required during cell differentiation [43], albeit a role for KMT2C monomethylation in a cardiac context is yet to be determined. Variants in *KMT2D* cause Kabuki syndrome, a rare congenital disease with multiple anomalies, in which around 70% of patients present with congenital heart defects, with a unique predilection for left-sided obstructive lesions [18,21,22,23,44]. Additional variants in *KMT2D* have been linked to various other congenital heart conditions [45,46,47,48,49]. Animal models of *KMT2D* mimic the cardiac phenotype seen in patients and have indicated a role in Notch signaling [18,19,20]. In addition, Kmt2D was shown to be essential for cardiac lineage differentiation of mouse embryonic stem cells (ESCs) by H3K4me3 activity at the promoter of cardiac-specific genes, thus promoting their expression [50]. Notably, that study did not investigate H3K4me1 or -me2 activity. Our findings show that in flies, Trr mediates both H3K4me1 and me2 methylation during fly heart development.

Combined, the data show that each of the COMPASS cores facilitates different methylation states during fly heart development. This implies that they act in concert and that each of the H3K4 methyltransferases—Set1, Trx, or Trr—is required for healthy heart development.

### 3.2. COMPASS Complex-Mediated Methylation during Heart Development and Disease

**Figure 7:**
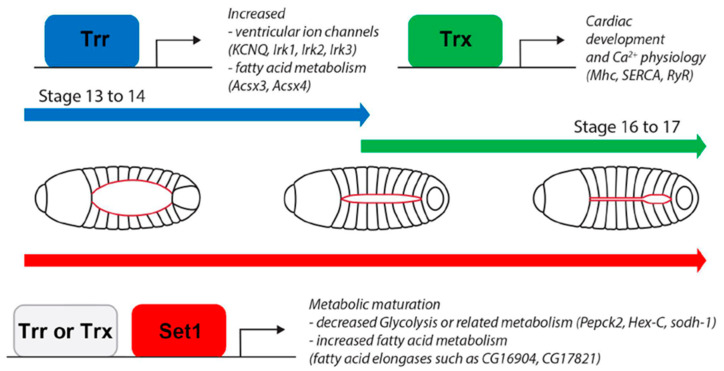
Model of *Set1-*, *trx-*, and *trr*-mediated *Drosophila* heart development. Graphical representation of proposed model for *SET domain containing 1* (*Set1*), *trithorax* (*trx*), and *trithorax-related* (*trr*)-mediated fly heart development; H3K4 methyltransferases and core subunits of the different COMPASS complexes. Stage refers to the established *Drosophila* developmental stages. Fly larvae demonstrate the developing heart, represented in red. At the early stages (stage 13–14), the heart progenitor cells migrate, and *Set1* and *trr* are active; during later stages (stage 16–17), the heart forms as a closed tube, *Set1* remains active, and *trx* takes over from *trr*.

Even though variants in COMPASS complex core subunits have been implicated in congenital heart disease, little is known about the underlying pathomechanisms or the roles of these core subunits in heart development. Here we showed that, like in mammals, in flies, the Set1, Trx, and Trr core units play essential roles in heart development. The transcriptional data further showed that the distinct methylation activity and preferred activity window for each COMPASS complex core subunit leads to the regulation of a diverse set of pathways. Several metabolic pathways were active early in development (Trr, Set1) and throughout (Set1), while muscle and heart differentiation processes were methylated during later stages of development (Trx) (Figure 7). Some pathways were shared, i.e., regulated by more than one COMPASS complex. However, we also identified several pathways, including those involved in ion transport and fatty-acyl-CoA metabolism, that were regulated oppositely by Trx- and Trr-mediated methylation. Ion transport genes are essential for muscle maturation [51], which is consistent with our data that showed Trx is required for heart development at late larval stages. The deletion of *Kmt2d* in mice identified similar pathways, including ion transport, which were regulated by Kmt2d methylation and caused structural and functional cardiac defects [18]. These findings indicate evolutionary conservation between *Drosophila* and mammals of the essential molecular mechanisms that regulate heart development.

Altogether, each of the COMPASS complexes mediates distinct methylation states and displays preferential times (early vs. later) of activity during fly heart development. However, we cannot rule out effects originating during pupal stages on adult methylation and gene expression as we focused on the earliest stages of heart development. Together, these findings strongly support the conservation of the COMPASS series complex in heart development from flies to mammals. Furthermore, our findings strengthen the value of *Drosophila* as a model system for both heart development and congenital heart diseases.

## 4. Materials and Methods

### 4.1. Drosophila Lines

*Drosophila* stocks were obtained from the Bloomington Drosophila Stock Center (Indiana University Bloomington, IN, USA). The following lines were used in this study: UAS-*Set1*-RNAi (ID 33704 and 40931), UAS-*trx*-RNAi (ID 31902 and 33703), and UAS-*trr*-RNAi (BDSC ID 29563 and 36916). Wildtype *w*^1118^ (BDSC ID 3605) flies were used in the crosses. The *twi*-Gal4 (BDSC ID 80574) and 4X*Hand*-Gal4 [25] drivers were used to express RNAi-based silencing (-IR) constructs in the heart starting early or late in larval development, respectively. *Hand*-GFP was previously generated by our team [52].

### 4.2. Live Imaging of Drosophila Larva

First instar larvae (*Hand*-GFP) were anesthetized by CO_2_ at room temperature. Fluorescence images of live anesthetized larvae were acquired using a ZEISS ApoTome.2 microscope with a 20X Plan-Apochromat 0.8 N.A. air objective (ZEISS, Jena, Germany). Images were obtained using ZEN blue edition (version 3.0) acquisition software. The whole heart was imaged by collecting Z-stacks. Since these images were used for cell counting, not the quantitation of fluorescence, the exposure time was modified for each genotype, if needed, to ensure the cardiomyocytes were clearly visible. ImageJ (version 1.49) (Schneider, Rasband, and Eliceiri 2012) was used for image processing. Six fly larvae were imaged per genotype, and representative images are shown in the figures.

### 4.3. Immunochemistry

*Drosophila* 5-day-old adult flies were dissected and fixed for 10 min in 4% paraformaldehyde in phosphate-buffered saline (1× PBS). Primary antibodies were incubated overnight at 4 °C in 2% bovine serum albumin (BSA; Sigma, St. Louis, MO, USA) with 0.1% Triton-X (Sigma, St. Louis, MO, USA) in 1× PBS. Following washes, secondary antibodies were incubated for 2 h at room temperature in 2% BSA (Sigma, St. Louis, MO, USA) with 0.1% Triton-X (Sigma, St. Louis, MO, USA) in 1× PBS. Alexa Fluor 555 phalloidin (Thermo Fisher, Waltham, MA, USA; A34055) was used at 1:1000 dilution. Mouse anti-Pericardin antibody (EC11; Developmental Studies Hybridoma Bank, Iowa City, IA, USA) was used at 1:500 dilution. Confocal imaging was performed using a ZEISS LSM900 microscope with a 63× Plan-Apochromat 1.4 N.A. oil objective (ZEISS, Jena, Germany). Images were obtained using ZEN blue edition (version 3.0) acquisition software. Segment A2 of the heart was imaged by collecting Z-stacks. Control groups were imaged first to establish the laser intensity and exposure time for the entire experiment. The exposure time was based on image saturation (at a set point of approximately 70% of maximum saturation) to enable the comparison of fluorescence intensity across all genotypes. ImageJ (version 1.49) (Schneider, Rasband, and Eliceiri 2012) was used for processing. Six fly larvae were imaged per genotype, and representative images are shown in the figures.

### 4.4. Heart Structural Analysis and Quantitation

*Drosophila* heart cardiac myofibril density, cardiomyocyte number, and Pericardin deposition were quantified as previously described [53]. For quantitative comparisons, we analyzed either six 1st instar larvae (female and male) for each genotype (*Hand*-GFP; *twi*-Gal4/4x*Hand*-Gal4 > UAS-*Set1*-IR/UAS-*trx*-IR/UAS-*trr*-IR) or six 5-day-old female adult flies for each genotype (*Hand*-GFP;*twi*-Gal4 > UAS-*Set1*-IR/UAS-*trx*-IR/UAS-*trr*-IR or control, *Hand*-GFP;*twi*-Gal4^+/−^). ImageJ software (version 1.49) [54] was used to process the images. The Z-stack projections were screened, and image levels containing cardiac myofibrils were selected for analysis while avoiding the ventral muscle layer that underlies the heart tube. The cardiac myofibril number was quantified by using the MyofibrilJ plugin for Fiji (version 1.53q) [55]. The entire heart region in segment A2 was selected using the freehand selection function in Fiji to count the number of cardiac myofibrils. For quantitation, cardiac myofibrillar density was calculated as the cardiac myofibril number divided by the size of the heart region. Cardiomyocyte numbers were manually counted in a standard-sized area in heart segment A2. Pericardin deposition was measured in heart segment A2 based on the fluorescence intensity. Cardiac myofibril density, cardiomyocyte number, and Pericardin deposition were each normalized to the values obtained in the control flies.

### 4.5. Lethality at Eclosion

Eclosion lethality is evidenced by the percentage of flies expressing an RNAi-based silencing construct (straight wings) (*twi*-Gal4 > UAS-*Set1*-IR/UAS-*trx*-IR/UAS-*trr*-IR) that fail to emerge as adults, relative to siblings that do not express the silencing construct (curly wings). The result is presented as a Mortality Index (MI) calculated as [((curly − straight)/curly) × 100]. At least 400 flies (female and male) were counted per genotype.

### 4.6. Adult Drosophila Survival Assay

*Drosophila* larvae were kept at 25 °C to induce UAS-transgene expression (*twi*-Gal4 > UAS-*Set1*-IR/UAS-*trx*-IR/UAS-*trr*-IR). Adult male flies were subsequently maintained in vials at 25 °C, with each vial containing 20 animals. Mortality was monitored every 48 h. One hundred flies were assayed per genotype.

### 4.7. Optical Coherence Tomography (OCT)

Cardiac function in adult *Drosophila* was measured using OCT. The system (Bioptigen) was built as described by the Biophotonics Group, Duke University, NC, USA [56,57]. Four-day-old flies (*twi*-Gal4 > UAS-*Set1*-IR/UAS-*trx*-IR/UAS-*trr*-IR) were anesthetized by carbon dioxide (CO_2_) for 3–5 min and females were preselected from each group. Each fly was gently placed on a plate with petroleum jelly (Vaseline) for immobilization with the dorsal aspect facing the OCT microscopy source and then rested for at least 10 min to ensure the fly was fully awake. For each genotype, 10 control and 10 RNAi-expressing flies were used. OCT was used to record the adult heart rhythm and heart wall movement in the same position, i.e., the cardiac chamber in the abdominal segment A2 of each fly. Each measurement was obtained in three different positions within the abdominal segment A2, and these were averaged to obtain the cardiac diameter for that fly. M-mode images recorded the heart wall movement during the cardiac cycle. ImageJ software (version 1.49) (Schneider, Rasband, and Eliceiri 2012) was used to process the images. The diastolic dimension and systolic diameter were processed, measured, and determined based on three consecutive heartbeats. The heart period was determined by counting the total number of beats that occurred during a 15-s recording and then dividing 15 by the number of beats.

### 4.8. Methylation Levels

Adult (5-day-old) female *Drosophila* (*Hand*-GFP;*twi*-Gal4 > UAS-*Set1*-IR/UAS-*trx*-IR/UAS-*trr*-IR or control, *Hand*-GFP;*twi*-Gal4^+/−^) were dissected and fixed for 10 min in 4% paraformaldehyde in phosphate-buffered saline (1× PBS). Primary antibodies were incubated overnight at 4 °C in 2% bovine serum albumin (BSA; Sigma, St. Louis, MO, USA) with 0.1% Triton-X (Sigma, St. Louis, MO, USA) in 1× PBS. Following washes, secondary antibodies were incubated for 2 h at room temperature in 2% BSA (Sigma, St. Louis, MO, USA) with 0.1% Triton-X (Sigma, St. Louis, MO, USA) in 1× PBS. We previously validated the anti-H3K4me1/2/3 antibodies in fly larval brain tissue (ventral nerve cord and central brain lobes), in which each showed clear expression in the neural nuclei [10]. Here, rabbit anti-H3K4me1 (Abcam, Cambridge, UK; ab8895), rabbit anti-H3K4me2 (Abcam, Cambridge, UK; ab7766), and rabbit anti-H3K4me3 (ActiveMotif, Carlsbad, CA, USA; 39159) were each used at a 1:2000 dilution, followed by Alexa Fluor 568 secondary antibodies at a 1:1000 dilution (Invitrogen, Waltham, MA, USA; A11011). Confocal imaging was performed using a ZEISS LSM900 microscope with a 63× Plan-Apochromat 1.4 N.A. oil objective (ZEISS, Jena, Germany). Images were obtained using ZEN blue edition (version 3.0) acquisition software. Segment A2 of the heart was imaged by collecting Z-stacks. Control groups were imaged first to establish the laser intensity and exposure time for the entire experiment. The exposure time was based on image saturation (at a set point of approximately 70% of maximum saturation) to enable the comparison of fluorescence intensity across all genotypes. ImageJ (version 1.49) (Schneider, Rasband, and Eliceiri 2012)was used for processing.

For quantitative comparison of the nuclear methylation levels, cardiac cells in segment A2 were analyzed. A single image typically captured four cells, equaling four nuclei. We obtained images from six flies for each genotype (RNAi or control), then used the mean fluorescent intensity in the nucleus of ~20 heart cells total (3/4 per fly) to determine the nuclear methylation level. Finally, nuclear methylation levels were normalized to the value obtained from the control flies. ImageJ (version 1.49) (Schneider, Rasband, and Eliceiri 2012) was used for processing. Representative images are shown in the figures.

### 4.9. RNA Extraction and Sequencing of Drosophila Cardiomyocytes

For each sample, hearts from 50 one-week-old adult female flies (*Hand*-GFP;*twi*-Gal4 > UAS-*Set1*-IR/UAS-*trx*-IR/UAS-*trr*-IR or control, *Hand*-GFP; *twi*-Gal4^+/−^) were manually dissected and collected in ice-cold TRIzol LS reagent (Thermo Fisher Scientific, Waltham, MA, USA). Then, RNA extraction was performed according to the instructions provided by the manufacturer (Direct-zol RNA Microprep, Zymo Research, Irvine, CA, USA). RNA quality and concentration were analyzed by agarose gel electrophoresis, the NanoDrop 8000 Spectrophotometer, and Agilent 2100 (Thermo Fisher Scientific, Waltham, MA, USA). More than 400 ng of total RNA for each sample was used in subsequent library preparation and sequencing.

Preparation of the RNA-Seq libraries and their sequencing were carried out by Novogene (Sacramento, CA, USA) using Illumina’s Nova-Seq platform with 150 bp read lengths. We mapped the short reads onto the *Drosophila melanogaster* reference genome release 6 [58], which we obtained from Ensembl [59]. We used STAR aligner (version 2.4.2a) [60] for short-read mapping with the following parameters: sjdbOverhang, 149; genomeSAindexNbases, 12; and alignIntronMax, 250,000. We used the gene annotation model from Ensembl release 100, which corresponds to FlyBase 6.28 [61]. For the gene and transcript level quantification, we used RSEM (version 1.2.22) [62] with default parameters. The differential expression analyses were carried out on the RSEM output with DESeq2 (version 1.34.0) [63] by comparing the RNAi fly samples to the control (one from this study, *Hand*-GFP; *twi*-Gal4^+/−^ and one from our previously published data (GSE173835), 4x*Hand*-Gal4 > *w*^1118^ one-week-old flies [64]). We used clusterProfiler (version 4.2.2) [65] for the gene ontology enrichment analysis.

### 4.10. Statistical Analysis

Statistical analysis was performed using PAST.exe software (version 2.17C) [Natural History Museum, University of Oslo (UiO), Oslo, Norway]. Values are presented as the mean along with the standard deviation (s.d). Data were first tested for normality using the Shapiro–Wilk test (a = 0.05). The Kruskal–Wallis H-test followed by Dunn’s test was used for comparisons between multiple groups (non-normal distributed data). For RNA-Seq, the Wald test was used, and *p*-values were correct for multiple testing using the Benjamini–Hochberg method. Statistical significance was defined as *p* (adjusted *p* for RNA-seq) < 0.05.

## Data Availability

All source data have been deposited in NCBI’s Gene Expression Omnibus and are accessible through GEO accession numbers: GSE240313 (this study), GSE173835 (control used for RNA-seq data analysis) [64], and GSE168774 (scRNA-seq of the developing fly heart) [26]. Other data and materials that support the findings of this study are available from the corresponding authors upon reasonable request.
